# Factors affecting cervical cancer screening and human papilloma virus vaccination among Middle Eastern refugee women in Türkiye: indicators for social workers and nurses

**DOI:** 10.1080/07853890.2025.2468261

**Published:** 2025-02-22

**Authors:** Yaser Snoubar, Baraka Abusafia, Zekiye Turan

**Affiliations:** a Social Sciences Department (Social Work Program), College of Arts and Sciences, College of Arts and Sciences, Qatar University, Doha, Qatar; b Department of Nursing, Faculty of Health Sciences, Sakarya University, Sakarya, Türkiye; c Nursing, Obstetric and Gynecological Nursing, Sakarya University, Sakarya, Türkiye

**Keywords:** Cervical cancer, cultural sensitivity, health promotion, human papilloma virus vaccination, refugee women

## Abstract

**Background:**

This study aimed to generate knowledge regarding factors affecting cervical cancer screening and human papillomavirus vaccination that can be used practically by social workers and nurses while working with Middle Eastern refugee women living in Türkiye.

**Methods and Results:**

This study involved the administration of a self-reported questionnaire to 227 female migrants. It identified sociodemographic variables, such as age, marital status, and education level, that influenced the participation of these women in preventive practices against cervical cancer. Despite their cultural beliefs, financial constraints, and language hurdles, the findings indicated that the likelihood of involvement in screening and vaccination for cervical cancer was greater among refugee women if they were well-informed and assisted.

**Conclusions:**

The results of this study suggest the necessity for specific training programs and improved initiatives for healthcare access to prevent cervical cancer among vulnerable groups. Healthcare providers can support preventive measures more effectively if these concerns are addressed with consideration of the sociocultural elements and language issues among Middle Eastern refugee women. Although some limitations associated with self-reported data collection methods may have introduced response bias, this study showed how social workers and nurses can act as facilitators to prevent cervical cancer.

## KEY MESSAGES

- Practical barriers such as language difficulties and financial constraints significantly hinder Middle Eastern refugee women’s access to cervical cancer screening and HPV vaccination services in Türkiye.
- Cultural and religious stigmas, though less significant than practical barriers, still prevent women from seeking cervical cancer screening and HPV vaccination, highlighting the need for culturally sensitive health promotion campaigns.
- Comprehensive educational programs are necessary to improve awareness and understanding of cervical cancer and HPV among refugee women, addressing misconceptions and emphasizing the importance of early screening and vaccination.

## 1. Introduction

Among human papillomavirus (HPV) strains, those causing cervical cancer rank among the most prevalent forms of the virus. Owing to its gradual progression, this type of cancer often remains undetected in its initial phases. According to the World Health Organization (WHO), cervical cancer is the second most frequently diagnosed cancer among women in less-developed regions worldwide. This underscores the critical requirement for enhanced health awareness, including public education on the causative elements and methods of transmission of the disease, as well as promoting a comprehensive understanding of HPV as the primary cause of this cancer. Furthermore, highlighting the importance of early HPV vaccination in young females is pivotal for reducing the incidence of cervical cancer [1].

HPV infection is recognized as a common sexually transmitted infection, affecting both sexes and leading to various conditions, such as recurrent respiratory papillomatosis, genital warts, and cancers of the cervix, vagina, penis, anus, and oropharyngeal regions [2,3]. Although cervical cancer is the condition most commonly associated with this infection, it is important to recognize the significant impact of HPV on morbidity and mortality in males [2]. HPV can also infect the male urogenital tract and have detrimental effects on natural and assisted fertility by binding strongly to the sperm head, resulting in decreased semen quality. Association between HPV and assisted reproductive technology program outcomes has been documented, with HPV infection of the semen negatively affecting the outcomes of these procedures. HPV vaccination has been proposed to improve viral clearance from the semen of infertile men, increasing the natural fertility of such affected couples [4].

In 2020, approximately 604,000 new cases of cervical cancer were diagnosed globally, ranking it as the fourth most common cancer among women worldwide. With a 90% mortality rate in 2018, predominantly in low-to middle-income countries, the urgency for action is clear. Thus, in 2020, the World Health Organization recommended a global cervical cancer eradication strategy that favors comprehensive treatment and prevention methods, such as the creation of awareness, social mobilization, vaccination, screening, and treatment [5]. The most effective method to prevent HPV-related diseases, such as cervical cancer, is through immunization. Further vaccine information dissemination campaigns and education are needed to promote changes in behavior among people [6,7]. HPV vaccines prevent infection and the development of precancerous lesions and contribute significantly to reducing the incidence of cervical cancer. Worldwide, screening for HPV-related lesions is performed using the Papanicolaou (Pap) cervical smear test and HPV testing. Finally, tertiary prevention aims to treat already diagnosed lesions with surgery, chemotherapy, radiotherapy, and immunotherapy [8]. According to the WHO’s ‘screen and treat approach,’ the treatment decisions should be based solely on a positive primary screening test. In contrast, their ‘screening, triage, and treatment approach’ suggests that the treatment decision should be based on a positive primary screening test that is followed by a positive secondary test (‘triage’ test), regardless of whether the diagnosis has been histologically confirmed [9]. Several advances have been made, including improvements in diagnostic tools, development of efficient vaccine formulations, and pre-invasive screening methodologies; however, further improvements are required, such as optimal vaccine dosing, knowledge on post-treatment vaccination timing, and awareness of home-based care in cases of HPV infection [10].

Legal challenges and disparities often affect healthcare delivery, particularly regarding migrants. Regardless of their immigration status, immigrants require equal service rendition, including access to preventive care, such as immunization and contraception, as these are key to maintaining public health [11,12]. However, this consideration has led some developed countries to rely heavily on vaccination alone as the primary preventive strategy against cervical cancer. Efforts must be made to bridge these developmental gaps across countries; otherwise, many people will continue to die because of a lack of knowledge on this subject, particularly within immigrant communities, where studies have shown that further work regarding HPV infection and immunization rates is required [13–15].

In Türkiye, the number of reported cases of cervical cancer has increased significantly over the years, particularly among Syrian refugees. Therefore, urgent action is required to ensure that knowledge is acquired at an early stage. The same prevalence rates between Turkish women and Syrian refugees in Türkiye indicate similar levels of understanding regarding its seriousness. Nevertheless, the focus should be on closing the gaps regarding understanding what constitutes effective cervical cancer screening among Syrians [12]. From a holistic point of view, cervical cancer should be targeted from all angles, including vaccination, provision of education, and creation of awareness, through equitable healthcare services targeting those who have been marginalized by society because they are either immigrants or belong to minority groups.

Thus, this study aimed to assess the awareness and concerns regarding HPV vaccination among refugee women from Middle Eastern countries who are residing in Türkiye. The study also examined how demographic factors determine the understanding of cervical cancer and HPV, while identifying barriers that hinder the effectiveness of immunization or screening campaigns. This study further aimed to add to current information regarding the prevention of cervical cancer, so that specific interventions can be developed for vulnerable populations. To meet these objectives, answers were sought to the following questions.

1. What prevents cervical cancer screening and HPV vaccination among Middle Eastern refugee women in Türkiye?
2. What is the extent of their knowledge regarding cervical cancer and risk factors?
3. How familiar are they with HPV infection and its potential consequences for health?
4. To what degree do they understand the HPV vaccine and its benefits?
5. How do factors, such as employment status, marital status, nationality, and educational attainment influence awareness and perceptions of cervical cancer, HPV, and access to screening and vaccination services?

## 2. Material and methods

### 2.1. Study design

This cross-sectional study collected data from female Middle Eastern refugees residing in Türkiye, using interviews conducted in Arabic, tailored to accommodate the language proficiency of the participants. The study protocol was approved by the local ethics committee, which ensured compliance with established research guidelines. The study obtained written informed consent from all participants, with ethical approval from the ethics committee of Sakarya University.

### 2.2. Setting and sample

Türkiye hosts around 5.1 million foreign nationals, 3.8 million of whom require international protection [16]. This study recruited female Middle Eastern refugees in Türkiye from migrant health centers. Refugee women aged 18 years and older who visited these centers between October 15 and December 15, 2023, were eligible.

The minimum number of participants required for this study was 149. The calculation was based on a 99% confidence level and a standard deviation of 0.05. It was assumed that 10% of potential respondents would drop out because they did not complete the survey. Thus, to ensure statistical significance and stability of the findings, we targeted at least 165 participants. In the recruitment phase, potential participants received a letter describing the study, and informing them that if they chose to participate, their identities would remain anonymous throughout the process of the study. Using these procedures, 227 refugee women from different Middle Eastern countries were recruited.

### 2.3. Questionnaire

The questionnaire used in this study was initially developed by Dona [1] and modified to address concerns linked to cervical cancer screening and HPV vaccination among Middle Eastern refugee women in Türkiye. The questionnaire development involved a rigorous process of expert scrutiny, cultural sensitivity assessment, and clarification in alignment with the goals of this study. The main purpose of this study was to identify barriers to healthcare access using specific questions that could adjust for language limitations or protect against incorrect information provision due to a lack of knowledge in certain areas. Consequently, this instrument was rigorously prepared to identify the issues influencing cervical cancer screening among Turkish Middle Eastern refugees. Its content spanned demographic data, obstacles to accessibility of healthcare, level of awareness of cervical cancer, rates of awareness of HPV, and understanding of HPV vaccines. The economic status of the participants was assessed and classified as ‘poor,’ ‘average,’ or ‘excellent,’ based on their self-reported income level, as compared to the average income within Türkiye [17]. This classification allowed for a clearer understanding of the economic context of the participants and its impact on their access to healthcare and on their behaviors.

### 2.4. Data collection procedures

For this study, the data collection strategy was designed such that confidentiality was protected, accuracy was guaranteed, and HPV vaccination and cervical cancer screening practices among refugee women from the Middle East living in Türkiye were holistically addressed. The procedure had several steps:

#### 2.4.1. Preparation phase

Appropriate tools were used for collecting data. A survey was developed that suited the cultural and linguistic intricacies of the respondent community. Bilingual social workers and nurses were recruited to support effective communication among people from different cultures, thereby facilitating an understanding of various perspectives held by the community.

#### 2.4.2. Questionnaire development

Closed- and open-ended questions were used to evaluate demographic information, knowledge of HPV and cervical cancer, barriers to healthcare access, and thoughts on screening for cervical cancer and vaccination against HPV. Social work and nursing professionals reviewed the questionnaire to ensure that it was appropriate, understandable, and culturally sensitive.

#### 2.4.3. Pilot phase

A pilot assessment with a selected target cohort verified the effectiveness of the questionnaire. Participant feedback led to minor revisions of the phrasing and structure to improve its comprehensibility.

#### 2.4.5. Ethical considerations and informed consent

Ethical approval for this study was obtained from the Sakarya University’s Social and Human Sciences Ethics Committee, which also approved the contracts, administrative procedures, and the content of the survey (Ref. E-61923333-050.99-294396) on October 12, 2023, as outlined in the protocols designed to protect human participants. The participants were informed about the study’s objectives, their right to withdraw, and the confidentiality and anonymity protocols established to protect their data. Each participant provided written informed consent before participating in the investigation. Furthermore, written consent for publishing these details was obtained from all individuals, with measures taken to anonymize personal information.

#### 2.4.6. Participant recruitment

The recruitment process aimed to obtain a diversified cohort from migrant health centers across Türkiye, considering factors such as nationality, age, and socioeconomic background. The recruitment strategy used postings in community centers, direct outreach in healthcare settings, and partnerships with local refugee aid organizations.

#### 2.4.7. Data gathering

Bilingual professionals conducted one-on-one interviews with participating refugee women, using the refined questionnaire. Interviews were organized at times and locations convenient for the participants, to foster a comfortable atmosphere, and lasted approximately 15–0 min. The interviewers presented questions neutrally and clarified queries, as required, to guarantee authentic and informed responses.

#### 2.4.8. Data analysis

Data analyses were performed using SPSS version 27 (IBM SPSS Inc., Armonk, NY, USA) for descriptive statistics and non-parametric tests, alongside SmartPLS software for analyses, including validity, reliability, and confirmatory factor analysis (CFA). SmartPLS was used to test the reliability and validity of the measurement model through internal consistency reliability, indicator reliability, convergent validity, and discriminant validity assessments. Additionally, SmartPLS was used for CFA, to analyze the structural relationships delineated in the model. The threshold for statistical significance was set at *p* < 0.05.

## 3. Results

### 3.1. Sample characteristics

The demographic characteristics of the participants are presented in Table 1.

**Table 1.** Demographic characteristics of the study participants.

Characteristic	Category	*n*	%
Nationality	Iraqi	46	20.26%
	Palestinian	15	6.61%
	Syrian	161	70.93%
	Yemeni	5	2.20%
Marital Status	Not Previously Married	61	26.87%
	Previously Married	166	73.13%
Educational Level	Less than University	82	36.12%
	University	128	56.39%
	Postgraduate	17	7.49%
Employment Status	Not Working	153	67.40%
	Working	74	32.60%
Economic Situation	Poor	32	14.10%
	Average	178	78.41%
	Excellent	17	7.49%
Religious Belief	Muslim	227	100%
Religiosity	Moderate	139	61.23%
	Strong	88	38.77%
Duration of Residence in Türkiye	Less than One Year	15	6.61%
	1–5 Years	102	44.93%
	5–10 Years	86	37.89%
	More than 10 Years	24	10.57%
Information on HPV and Vaccination	No	172	75.77%
	Yes	55	24.23%

### 3.2. Barriers to healthcare access and cultural attitudes

This subsection examines barriers to healthcare access and cultural attitudes towards cervical cancer screening and HPV vaccination.

Table 2 illustrates the mean scores and standard deviations (SD) for items related to perceived barriers. Regarding practical and accessibility issues, the participants reported moderate difficulty in accessing and benefiting from health services, with language barriers cited as significant (mean = 2.95, SD = 0.75). Concerns regarding the affordability of HPV vaccination were also notable (mean = 2.69, SD = 0.74). Regarding cultural and religious stigma, the results indicated a lower perceived stigma associated with cervical cancer screening and vaccination, with the strongest stigma being the belief that cancer is ‘God’s will’ and is not preventable through vaccination (mean = 2.19, SD = 0.75). The least endorsed stigma was the religious inadvisability of screening for cervical cancer (mean = 1.60, SD = 0.66).

**Table 2.** Perceived barriers to cervical cancer screening and HPV vaccination.

Code	Items	Mean	SD
	Practical and Accessibility Issues	2.63	0.60
BSV1	As a refugee, it is not easy for me to access and benefit from health services	2.45	0.84
BSV2	The language barrier is a major problem in obtaining such services.	2.95	0.75
BSV3	My living conditions as a refugee do not allow me to get cervical cancer screening	2.43	0.77
BSV4	I think I cannot afford the HPV vaccination.	2.69	0.74
	Cultural and Religious Stigmas	1.87	0.57
BSV5	It is shameful for a woman to go to a health center for an examination and a cervical smear.	1.88	0.83
BSV6	It is a shame that I received the HPV vaccination.	1.83	0.74
BSV7	I believe that cancers are God’s will, and that vaccination cannot prevent them.	2.19	0.75
BSV8	I think it is not religiously recommended to screen for cervical cancer.	1.60	0.66

#### 3.2.1. Knowledge of cervical cancer: risk factors and screening

This section examines the level of knowledge among Middle Eastern refugee women regarding the risk factors associated with cervical cancer and their understanding of the disease screening process and progression. The level of knowledge of refugee women regarding the HPV vaccine, including its effectiveness, dosage, administration, and the necessity of ongoing screening post-vaccination was evaluated.

Table 3, using the coding taken from the Elsevier Vaccine Journal, presents mean scores and SDs regarding understanding of cervical cancer risk factors and screening methods. Concerning risk factors and prevention, participants scored the highest on the belief that cervical cancer was preventable (mean = 3.63, SD = 1.06). In contrast, the lowest score was related to the understanding that early childbirth increases the risk of cervical cancer (mean = 2.85, SD = 1.21).

**Table 3.** Knowledge regarding cervical cancer, HPV, and HPV vaccine of refugee women.

Refugee Women’s Knowledge of Cervical Cancer Risk Factors and Screening			
Code	Items	Mean	SD
	Knowledge of Risk Factors and Prevention	3.27	0.78
KCC1	Smoking increases the risk of cervical cancer.	3.36	1.13
KCC2	Birth control pills increase the risk of cervical cancer.	3.22	1.20
KCC3	Giving birth at an early age increases the risk of cervical cancer.	2.85	1.21
KCC4	Weak immunity increases the risk of cervical cancer.	3.48	1.06
KCC5	There are no symptoms of cervical cancer in its early stages.	3.11	1.10
KCC6	Cervical cancer can be prevented.	3.63	1.06
KCC10	Viral infections cause cervical cancer.	3.23	1.14
KCC11	Genetic factors cause cervical cancer.	3.31	1.14
	Understanding of Screening and Progression	3.35	0.98
KCC7	Cervical cancer cannot be cured in its advanced stages	2.84	1.20
KCC8	Cervical cancer can be cured in its advanced stages.	3.15	1.30
KCC9	Cervical cancer can affect women of all age groups.	3.43	1.23
KCC12	The Pap smear test must be repeated every 3–5 years	3.68	1.17
KCC13	A Pap smear test is performed to test for cervical cancer	3.63	1.25
Knowledge of HPV Effects, Impact, and Transmission			
Code	Items	Mean	SD
	Effects and Impact of HPV	3.33	0.93
KHP1	HPV causes cervical cancer	3.39	1.00
KHP6	HPV is treatable	3.50	1.03
KHP7	HPV affects men and women	3.03	1.35
KHP8	HPV can cause genital warts	3.41	1.10
	Transmission and diversity of HPV	3.07	0.93
KHP2	Cervical cancer cannot be cured in its advanced stages	3.00	0.98
KHP3	Cervical cancer can be cured in its advanced stages.	3.11	1.11
KHP4	The Pap smear test must be repeated every 3–5 years	3.26	1.25
KHP5	A Pap smear test is performed to test for cervical cancer	2.92	1.26
Knowledge of the HPV Vaccine			
Code	Items	Mean	SD
	Knowledge About the HPV Vaccine	3.37	0.73
KVA1	Vaccination at an early age is more effective in preventing cervical cancer	3.80	0.95
KVA2	Women can get the HPV vaccine at any stage of their life	3.33	1.05
KVA3	The dose is taken through a syringe	3.41	1.03
KVA4	The number of doses ranges from 2–3 doses	3.32	0.96
KVA5	It is preferable to vaccinate girls before the age of 12 years	2.66	1.26
KVA6	The vaccine protects against cervical cancer	3.51	1.08
KVA7	Pap smears must be performed continuously after vaccination	3.55	1.06

Regarding screening practices, the participants demonstrated strong knowledge of the importance of regular Pap smear tests every 3–5 years (mean = 3.68, SD = 1.17). However, respondents demonstrated less certainty regarding the curability of advanced-stage cervical cancer, as indicated by a lower score (mean = 2.84, SD = 1.20), suggesting varying beliefs among participants.

Table 3 also shows the perceptions of the impact of HPV, including its effects, transmission, and diversity. Participants demonstrated a generally high level of consensus among the participants regarding the treatability of HPV (mean = 3.50, SD = 1.03) and its association with genital warts (mean = 3.41, SD = 1.10). Participants moderately agreed that HPV affects both sexes (mean = 3.03, SD = 1.35), highlighting the recognition of HPV as a concern for males and females.

Regarding the transmission and diversity of HPV, some uncertainty appeared to exist regarding the curability of advanced-stage cervical cancer, with mean scores centering around the midpoint of the scale (mean = 3.00, SD = 0.98 for non-curability and mean = 3.11, SD = 1.11 for curability). Participants also moderately acknowledged the importance of regular Pap smear tests (mean = 3.26, SD = 1.25).

Finally, Table 3 shows the level of knowledge regarding the HPV vaccine, with high agreement on the efficacy of early age vaccination in preventing cervical cancer (mean = 3.80, SD = 0.95). Recognition of the availability of the vaccine at any life stage (mean = 3.33, SD = 1.05) and requirement for ongoing post-vaccination Pap smear tests (mean = 3.55, SD = 1.06) were also notable. However, some uncertainty regarding the preference for vaccinating girls prior 12 years of age was present, as this item received the lowest mean score (mean = 2.66, SD = 1.26).

### 3.3. Reliability

The reliability of the survey items was determined by examining standardized factor loadings. The literature suggested a loading of 0.70 as optimal; however, for exploratory studies, a minimum threshold of 0.50 is deemed acceptable [18]. The findings, as detailed in Table 4, demonstrated that all items achieved factor loadings above the exploratory threshold, confirming convergent validity at the item level.

**Table 4.** Measurement model results.

Factor	Items	SL	*α*	CR	AVE
Practical and Accessibility Issues	BSV1	0.74	0.89	0.72	0.76
	BSV2^a^	–			
	BSV3	0.95			
	BSV4	0.91			
Cultural and Religious Stigmas	BSV5	0.92	0.87	0.95	0.72
	BSV6	0.90			
	BSV7	0.77			
	BSV8	0.80			
Knowledge of Risk Factors and Prevention	KCC1	0.78	0.89	0.90	0.58
	KCC2	0.81			
	KCC3	0.81			
	KCC4	0.77			
	KCC5	0.70			
	KCC6	0.72			
	KCC10	0.79			
	KCC11	0.67			
Understanding of Screening and Progression	KCC7^a^	–	0.80	0.82	0.71
	KCC8^a^	–			
	KCC9	0.83			
	KCC12	0.83			
	KCC13	0.86			
Effects and Impact of HPV	KHP1	0.82	0.82	0.83	0.65
	KHP6	0.72			
	KHP7	0.80			
	KHP8	0.87			
Transmission and diversity of HPV	KHP2	0.81	0.77	0.77	0.59
	KHP3	0.76			
	KHP4	0.69			
	KHP5	0.80			
Knowledge About the HPV Vaccine	KVA1	0.81	0.86	0.86	0.54
	KVA2	0.70			
	KVA3	0.72			
	KVA4	0.72			
	KVA5	0.70			
	KVA6	0.75			
	KVA7	0.75			

SL: Standard Loadings; α: Cronbach’s alpha; CR: Composite Reliability; AVE: Average Variance Extracted.

^a^ Items with SL less than 0.5 were omitted. ‘Standard Loadings’ refers to the factor loadings in confirmatory factor analysis, indicating the correlation between the observed variables and their underlying latent constructs.

Construct reliability was evaluated using Cronbach’s alpha and composite reliability measures. Consistent with Taber’s (2017) [19] recommendation, values above 0.70 indicate acceptable reliability. As shown in Table 4, Cronbach’s alpha and composite reliability indices surpassed this benchmark, verifying the reliability of the constructs in the study.

### 3.4. Validity

Convergent validity was examined by assessing the average variance extracted (AVE). Following the criteria of Henseler et al. [20], AVE values should ideally exceed 0.50. The AVE results, shown in Table 4, confirmed that all the constructs met and exceeded the minimum standard, indicating robust convergent validity.

Discriminant validity was measured using the Heterotrait–Monotrait ratio of correlations (HTMT). As shown in Table 5, none of the HTMT values surpassed the critical threshold of 0.85, thereby supporting the discriminant validity of this model. These results confirmed the distinctiveness of the constructs within the proposed research framework.

**Table 5.** Discriminant validity assessment using HTMT ratio of correlations.

HTMT Ratio of Correlation	(A)	(B)	(C)	(D)	(E)	(F)	(G)
(A) Cultural and religious stigmas							
(B) Effects and impact of HPV	0.18						
(C) HPV vaccine	0.12	0.69					
(D) Knowledge of risk factors and prevention	0.07	0.59	0.43				
(E) Practical and accessibility issues	0.28	0.11	0.07	0.14			
(F) Transmission and diversity of HPV	0.07	0.66	0.47	0.47	0.08		
(G) Understanding of screening and progression	0.16	0.41	0.27	0.25	0.07	0.33	

### 3.5. Comparative analysis based on employment status

The Mann–Whitney U test was used to investigate how employment status might affect perceptions and knowledge regarding HPV and cervical cancer. Analysis of the mean ranks between employed and unemployed participants revealed no significant differences in any of the examined areas (*p* > .05 for all). For instance, in ‘Practical and Accessibility Issues,’ unemployed participants had a slightly higher mean rank (117.77) than that of those who were employed (106.20); however, the difference was not statistically significant (*U* = 5,084, z = −1.26, *p* = .21). The lack of significant disparity was consistent across other factors, such as ‘Cultural and Religious Stigmas’ and ‘Knowledge of Risk Factors and Prevention,’ indicating that employment status did not significantly impact the level of knowledge or act as perceived barriers towards HPV and cervical cancer knowledge and perceptions.

### 3.6. Influence of marital status on perceptions and knowledge

The study also explored whether marital status affected understanding of or acted as perceived obstacles to knowledge and perceptions of HPV, the vaccine, and cervical cancer. Using the Mann–Whitney U test to compare married participants with unmarried participants, the findings showed no significant difference in knowledge or perceptions across any of the factors (*p* > .05 for all). The largest mean rank difference was observed in ‘Understanding of Screening and Progression’ between unmarried (123.64) and married women (110.46); however, it was not statistically significantly different (*U* = 4475, z = −1.35, *p* = .18). Other areas, such as ‘Practical and Accessibility Issues,’ ‘Cultural and Religious Stigmas’, ‘Knowledge of Risk Factors and Prevention,’ ‘Effects and Impact of HPV,’ ‘Transmission and Diversity of HPV,’ and ‘Knowledge About HPV Vaccine’ also showed no significant disparities, indicating that marital status did not markedly affect knowledge or perceived barriers in these health areas.

### 3.7. Cross-national comparison of HPV-related knowledge and perceptions

This section explores the variance in knowledge and perceptions regarding HPV and cervical cancer among participants, grouped by nationality of the refugee women.

Table 6 presents the findings of the Kruskal–Wallis H tests, comparing the mean response ranks regarding HPV and cervical cancer-related factors among the Iraqi, Palestinian, Syrian, and Yemeni groups. The results show a marginally significant distinction in ‘Practical and Accessibility Issues’ across nationalities (*H* = 7.80, df = 3, *p* = .05), with Iraqis experiencing the greatest barriers. In contrast, ‘Cultural and Religious Stigmas’ showed no significant difference across nationalities (*H* = 1.89, df = 3, *p* = .60).

**Table 6.** Differences in knowledge and perceptions by nationality.

Factors	Nationality	*N*	Mean Rank	Kruskal-Wallis H	df	*p*-value
Practical and Accessibility Issues	Iraqi	46	134.47	7.80	3	.05
	Palestinian	15	124.43			
	Syrian	161	108.31			
	Yemeni	5	77.70			
Cultural and Religious Stigmas	Iraqi	46	125.54	1.89	3	.60
	Palestinian	15	112.70			
	Syrian	161	111.14			
	Yemeni	5	103.80			
Knowledge of Risk Factors and Prevention	Iraqi	46	112.59	0.32	3	.96
	Palestinian	15	118.33			
	Syrian	161	114.43			
	Yemeni	5	100.20			
Understanding of Screening and Progression	Iraqi	46	101.70	2.62	3	.45
	Palestinian	15	120.10			
	Syrian	161	116.21			
	Yemeni	5	137.60			
Effects and Impact of HPV	Iraqi	46	100.53	4.79	3	.19
	Palestinian	15	125.27			
	Syrian	161	118.01			
	Yemeni	5	75.10			
Transmission and diversity of HPV	Iraqi	46	111.34	0.97	3	.81
	Palestinian	15	125.83			
	Syrian	161	114.23			
	Yemeni	5	95.50			
Knowledge About the HPV Vaccine	Iraqi	46	100.88	11.56	3	.01
	Palestinian	15	155.80			
	Syrian	161	115.56			
	Yemeni	5	59.10			

Crucially, ‘Knowledge About HPV Vaccine’ differed significantly among refugee groups (*H* = 11.56, df = 3, *p* = .01) and suggested that Palestinian refugee women possessed greater awareness or knowledge about the vaccine. Other measured factors, such as ‘Knowledge of Risk Factors and Prevention’ and ‘Effects and Impact of HPV’ showed no significant national variability (*p* > .05 for all).

### 3.8. Impact of education level on HPV-related knowledge and perceptions

This analysis aimed to determine whether the level of education affected knowledge and perceptions of HPV, the vaccine, and cervical cancer among refugee women.

Table 7 summarizes the findings of the Kruskal–Wallis H tests on the impact of education level (less than university, university, and postgraduate) on HPV and cervical cancer-related factors. Significant variances emerged in ‘Practical and Accessibility Issues’ (*H* = 7.29, df = 2, *p* = .03) and ‘Effects and Impact of HPV’ (*H* = 7.63, df = 2, *p* = .02), showing that education level affected perceptions in these domains, with women having less than a university education facing more barriers and differing in their understanding of the effects of HPV compared to those who were more educated.

**Table 7.** Differences in knowledge and perceptions by education level.

Factors	Education Level	*N*	Mean Rank	Kruskal-Wallis H	df	*p* value
Practical and Accessibility Issues	Less than University	82	124.71	7.29	2	.03
	University	128	111.76			
	Postgraduate	17	79.24			
Cultural and Religious Stigmas	Less than University	82	126.77	6.27	2	.04
	University	128	109.03			
	Postgraduate	17	89.82			
Knowledge of Risk Factors and Prevention	Less than University	82	113.23	0.16	2	.92
	University	128	113.69			
	Postgraduate	17	120.06			
Understanding of Screening and Progression	Less than University	82	110.85	0.33	2	.85
	University	128	115.45			
	Postgraduate	17	118.26			
Effects and Impact of HPV	Less than University	82	100.11	7.63	2	.02
	University	128	119.19			
	Postgraduate	17	141.91			
Transmission and diversity of HPV	Less than University	82	111.52	0.31	2	.86
	University	128	116.10			
	Postgraduate	17	110.15			
Knowledge About the HPV Vaccine	Less than University	82	111.15	0.25	2	.88
	University	128	115.43			
	Postgraduate	17	117.03			

Differences in ‘Cultural and Religious Stigmas’ (*H* = 6.27, df = 2, *p* = .04) indicated variations in cultural and religious views according to educational level. However, ‘Knowledge of Risk Factors and Prevention,’ ‘Understanding of Screening and Progression,’ ‘Transmission and Diversity of HPV,’ and ‘Knowledge About HPV Vaccine’ showed no significant impact of education (*p* > .05 for all).

### 3.9. Association between economic situation and HPV-related knowledge and perceptions

This section evaluates how the economic situation of refugee women may influence their knowledge and perceptions of HPV, the vaccine, and cervical cancer.

Table 8 shows the results of the Kruskal–Wallis H tests examining mean rank discrepancies based on economic status, categorized as poor, average, or excellent. A notable contrast was observed in ‘Practical and Accessibility Issues’ (*H* = 34.02, df = 2, *p* < .001), with individuals in poor economic circumstances perceiving significantly higher barriers (mean = 163.48). Similarly, ‘Effects and Impact of HPV’ demonstrated a statistically significant difference (*H* = 6.20, df = 2, *p* = .04), implying that economic status may influence perceptions of the impact of HPV. However, no significant variations were detected in terms of ‘Cultural and Religious Stigmas,’ ‘Knowledge of Risk Factors and Prevention,’ ‘Understanding of Screening and Progression,’ ‘Transmission and Diversity of HPV,’ and ‘Knowledge About HPV Vaccine’ (*p* > .05 for all).

**Table 8.** Economic situation and its relation to HPV knowledge and perceptions.

Factors	Economic Situation	*N*	Mean Rank	Kruskal-Wallis H	df	*p* value
Practical and Accessibility Issues	Poor	32	163.48	34.02	2	<.001
	Average	178	110.90			
	Excellent	17	53.29			
Cultural and Religious Stigmas	Poor	32	128.50	4.58	2	.10
	Average	178	113.99			
	Excellent	17	86.76			
Knowledge of Risk Factors and Prevention	Poor	32	126.53	1.63	2	.44
	Average	178	111.19			
	Excellent	17	119.79			
Understanding of Screening and Progression	Poor	32	128.09	3.36	2	.19
	Average	178	109.86			
	Excellent	17	130.79			
Effects and Impact of HPV	Poor	32	112.19	6.20	2	.04
	Average	178	110.71			
	Excellent	17	151.88			
Transmission and diversity of HPV	Poor	32	124.77	2.61	2	.27
	Average	178	110.40			
	Excellent	17	131.38			
Knowledge About the HPV Vaccine	Poor	32	113.06	0.01	2	.99
	Average	178	114.24			
	Excellent	17	113.26			

## 4. Discussion

This study on factors affecting cervical cancer screening and HPV vaccination among Middle Eastern refugee women in Türkiye provided pivotal insights that may be particularly valuable for social workers and nurses involved in health promotion and disease prevention in this vulnerable population. The data revealed critical barriers to healthcare access and highlighted cultural attitudes, while indicating the current manner of healthcare utilization among these women. The discovery that practical and logistical barriers, such as language and affordability, pose significant obstacles to accessing health services, including cervical cancer screening and HPV vaccination, suggests the need for targeted interventions. Strategies to enhance accessibility include linguistic support, information delivery in various languages, financial aid, and free vaccinations. Moreover, fewer cultural and religious stigmas impeded cervical cancer screening and vaccination than did the practical barriers experienced by the participants, which indicates a promising direction for health education. In addition, a deeper understanding of gender roles within these cultural and religious contexts may assist in identifying the factors that hinder and facilitate access of women to healthcare, highlighting the influence of these dynamics on health-related decisions.

Health promotion campaigns must be culturally sensitive. They should respect cultural diversity and should account for the religious and gender considerations of the targeted communities. To do so, individuals in these communities should be actively involved in designing and disseminating programs to ensure effectiveness and cultural appropriateness [14,21,22]. Such approaches would strengthen acceptance and engagement among the targeted groups. Well-targeted health promotion initiatives that understand these sociocultural aspects are often successful [23].

Prevention rather than treatment methods are preferred [24,25]. Therefore, considering the responses of the participants, comprehensive educational programs are needed because individuals have different levels of knowledge regarding cervical cancer and HPV. Such educational programs should involve clarifying misconceptions about the risk factors associated with the importance of early screening and the benefits of vaccination [18]. Thus, communication quality could be improved by addressing the specific concerns that women raise through such educational programs.

Moreover, the findings from this study on differences between nationalities regarding heath beliefs and knowledge in the refugee populations indicate that public health policies in Türkiye should be tailored to a wider Middle Eastern refugee community and in terms of specific national groups [26,27]. Finally, the connection between economic situations and perceived barriers to healthcare access about HPV emphasizes the need for policies regarding socioeconomic determinants of health. Gender sensitivity is crucial in such policy formulation [28,29].

## 5. Limitations and indicators for future studies

The cross-sectional nature and use of self-reporting in the survey may have led to limitations. The present conditions of the refugees, recollections of their homelands, and expectations for the future may introduce bias toward positive or negative replies. Additionally, self-reporting may not adequately represent the knowledge and habits regarding cervical cancer screening and HPV vaccination. Future studies should evaluate and enhance the survey results using qualitative and quantitative methodologies, such as focus groups or in-depth interviews.

The participants were limited to Middle Eastern refugee women who accessed migrant health services in Türkiye, which may not reflect most refugees. People who visited health facilities may have greater awareness and be more receptive to healthcare services than those who did not, which may constitute a selection bias. Therefore, our results cannot be generalized to all refugee groups or to all Middle Eastern refugee women in Türkiye. A larger and more varied cohort of refugee women, including those who do not use health facilities, would assist in understanding the barriers to, and facilitators of, cervical cancer prevention.

The applicability of the results of this study to refugee groups beyond Türkiye is limited. Refugees may have different cultural, religious, and social dynamics that affect their health practices and access to treatment. Thus, although this study provides useful insights, its models and conclusions should be extrapolated cautiously. Working with local health experts, community leaders, and refugees from different countries may assist in adapting treatments to various refugee populations.

Finally, understanding these limitations is essential for evaluating the results of the present study and directing future studies. Understanding and resolving these issues will assist in designing more effective future studies that are culturally appropriate and have generally applicable techniques, to increase cervical cancer screening and HPV vaccination rates among refugees.

## 6. Conclusion

This study highlights the importance of increasing public awareness of cervical cancer screening and HPV vaccination among female refugees in Türkiye, which reinforces the importance of social work and nursing interventions necessary for such communities. The major challenges identified included limited access to healthcare, current cultural practices, and gaps in knowledge. The results showed that cooperation among healthcare providers, policymakers, and social workers is needed in designing health promotion programs that incorporate cultural aspects. In addition, to a change in systems to ensure fair distribution of health services is necessary, which will lead to community participation in effective cervical cancer prevention programs and an increased number of easy-to-reach clinics.

This study stressed the requirement for a comprehensive strategy that addresses sociocultural, economic, and systemic hurdles to the lack of involvement of refugee women in cervical cancer screening and HPV vaccination. In addition, further studies into the elements influencing gender-sensitive patterns of care facility use, such as gendered familial roles, are urgently needed. For this reason, communication strategies must be developed to overcome culture-bound barriers, including strategies aimed at improving the health outcomes of displaced populations among individuals stigmatized by gender inequality or marginalized through restrictive practices.

## Data Availability

The data that support the findings of this study are not publicly available because this information that could compromise the privacy of research participants. The data will be shared on reasonable request to the corresponding author.
